# Pseudorabies Virus UL41 Hijacks IFN Response via JAK/STAT Pathway While Cellular TRIM21 Blocks it Through K48 Ubiquitination

**DOI:** 10.1155/tbed/3468674

**Published:** 2025-11-25

**Authors:** Xue Li, Jiawei Zheng, Guoqing Zhang, Peiheng Li, Mengzhen Dong, Quan Liu, Linzhu Ren

**Affiliations:** ^1^College of Animal Sciences, State Key Laboratory for Diagnosis and Treatment of Severe Zoonotic Infectious Diseases, Jilin University, Changchun, China; ^2^The First Hospital of Jilin University, Jilin University, Changchun, China; ^3^Key Lab for Zoonoses Research, Ministry of Education, Jilin University, Changchun, China

**Keywords:** JAK/STAT pathway, pseudorabies virus (PRV), tripartite motif protein 21 (TRIM21), ubiquitination, UL41/virion host shutoff (vhs)

## Abstract

Pseudorabies virus (PRV), a significant pathogen that infects various animals, including pigs, encodes multiple proteins that participate in host–pathogen interactions. This study investigates the mechanisms by which PRV evades host immune responses, with a particular focus on the role of the UL41 protein and its interactions with host factors. We found that PRV infection modulates the interferon (IFN) signaling pathway, suppressing the expression of IFN-β and downstream antiviral factors while upregulating IFN-α. However, the direct role of UL41 in IFN-α upregulation remains to be elucidated. The PRV UL41 protein was shown to directly target the JAK/STAT pathway, binding to specific motifs, such as the conserved sequences KUUUCY and CSDGGA, in the untranslated region (UTR) of key mRNAs and degrading them, thereby inhibiting IFN-I signal transduction. Simultaneously, the UL41 can interact with host proteins, such as poly(A) binding protein (PABPC1) and host restriction factor tripartite motif protein 21 (TRIM21). Additionally, we discovered an antagonistic relationship between PRV UL41 and TRIM21. TRIM21, acting as an E3 ubiquitin ligase, binds to UL41 through its SPRY/PRY domain and mediates the degradation of the protein via the K48-ubiquitin-proteasome pathway. This interaction modulates the JAK/STAT pathway, with TRIM21 counteracting the inhibitory effect of UL41. In addition, the residue F78 within PRV UL41 is crucial for modulating mRNA and protein binding and ribonuclease (RNase) function, facilitating interactions with target proteins such as PABPC1 and TRIM21, and inhibiting the JAK/STAT pathway. These findings enhance our understanding of PRV pathogenesis and provide potential targets for developing novel antiviral strategies.

## 1. Introduction

Pseudorabies virus (PRV; *Suid alphaherpesvirus 1*) exhibits a high degree of infectivity and pathogenicity within the swine population. Although pigs serve as the natural reservoir for PRV, the virus can infect a diverse range of mammals, including felines, canines, wolves, bovines, and even humans [1, 2], manifesting a spectrum of clinical signs, including intense pruritus and neurological dysfunction [3–6]. The extensive host range of PRV not only significantly enhances the risk of its transmission but also complicates the development of effective control (Con) and prevention strategies [7–9].

The UL41 protein, also known as the virion host shutoff (vhs) protein, is a tegument component within the *A*lphaherpesvirus family, which includes PRV, equine herpesvirus-1 (EHV-1), varicella-zoster virus (VZV), and herpes simplex virus (HSV) [10, 11]. This small protein has a molecular mass of around 58 kDa and adopts a globular conformation featuring a central hydrophobic core and several surface loops. Its C-terminal domain contains phosphorylation sites and a nuclear localization signal, both of which are crucial for regulating protein localization and function. In contrast, the N-terminal region is involved in RNA binding, interacting with both single-stranded RNA (ssRNA) and double-stranded RNA, as well as other viral and host proteins, and plays a significant role in the replication and pathogenicity of herpesviruses [10, 12–17]. Deletion or mutation of the *UL41* gene leads to a marked reduction in viral replication and attenuation of virulence in animal models [12–14].

In HSV-1, the vhs protein preferentially cleaves the 5′ end of mRNA with a cap or internal ribosome entry site (IRES) through its ribonuclease (RNase) activity [18–21]. It accomplishes this by binding to eukaryotic translation initiation factor 4A (eIF4A), eIF4F, and eIF4H during translation initiation and translocating the poly(A) binding protein (PABPC1) to the nucleus, consequently inhibiting host protein synthesis [18–21]. PRV UL41 can cleave ssRNA, mRNA with a cap and poly-A tail, and rRNA in vitro [22, 23]. The preferential sequences in mRNA are IRES or sequences downstream of the IRES [22]. Notably, the 5′-to-3′ RNase activity of PRV UL41 can be augmented by translation initiation factors such as eIF4H and eIF4B but reduced by point mutations at residues, including D152, D169, D171, D172, D173, P343, P345, L352, and W356 within the UL41, indicating its cardinal role in modulating viral and host RNA metabolism during infection. However, the RNase activity of the UL41/vhs proteins of HSV-1 and PRV depends on Mg^2+^ [23, 24], highlighting the complexity and significance of the UL41 function within the viral life cycle. In other alphaherpesviruses, such as VZV and EHV-1, the UL41/vhs protein also participates in mRNA and rRNA degradation, with its functions and mechanisms differing from those in HSV-1 and PRV [16]. Besides, it has been reported that the ability of UL41 to degrade host mRNAs facilitates the translation of viral mRNAs, thereby enhancing viral protein synthesis and replication [18, 19]. In addition, the UL41 protein has also been implicated in regulating viral gene expression, particularly during the transition from latent to lytic infection [15]. For example, HSV-1 vhs degrades viral mRNA [16] and interacts with viral proteins such as VP16 [18, 19, 25–28], thereby modulating viral gene expression and replication.

During virus infection, pattern recognition receptors detect viral components, triggering the synthesis and release of type I interferons (IFNs-I) through the JAK/STAT signaling pathway or other mechanisms [29, 30]. The binding of IFN-I to its corresponding cell surface receptors activates antiviral factors, such as interferon-stimulated genes (ISGs), which impede viral replication and spread. Studies have shown that HSV-1 UL41 participates in the virus's evasion of the host immune response by suppressing the synthesis of cytokines and interferons (IFNs) [14, 16]. During PRV infection, the UL41 protein exerts immunosuppressive effects by blocking the nuclear translocation of NF-κB and IRF3 while inducing the expression of TNF-α [13, 31]. These features allow the virus to escape the host immune system, underscoring the critical role of the UL41 protein in PRV pathogenesis and its potential as a therapeutic target.

To date, significant progress has been made in understanding the structure, function, and mechanism of the HSV-1 vhs protein. However, several key aspects remain poorly understood. The dynamic behavior of the UL41/vhs protein during the viral infection cycle and its detailed interactions with other viral and host proteins also need further investigation. This study focuses on PRV UL41 and examines the interplay between the PRV UL41 and IFN signaling pathways, as well as cellular mechanisms that counteract UL41-mediated immunosuppression, aiming to provide valuable insights for the development of innovative antiviral strategies. The zoonotic potential underscores the need to understand PRV's immune evasion mechanisms in both porcine and human cells. Therefore, this study addresses a gap by using both PK-15 (porcine kidney cell line) and HEK293 (human kidney cell line) cell models.

## 2. Materials and Methods

### 2.1. Cells and Virus

Cell lines, including HEK293 cells and porcine cells (PK-15 cells), were previously purchased from Shanghai Tong Wei Biological Technology Co., Ltd. (China), and cell culture was performed according to our previous protocol [32]. Briefly, HEK293 and HEK293T cell lines were cultured in Dulbecco's Modified Eagle's Medium (DMEM, Gibco, USA) supplemented with 10% fetal bovine serum (FBS, MeilunBio, Liaoning, China). PK-15 and Vero cells were cultured in DMEM (Gibco, USA) supplemented with 5% FBS (MeilunBio, Liaoning, China). All cells were maintained at 37°C in a standard, humidified incubator with 5% CO_2_.

PRV JL-21 strain (a field isolate with moderate virulence, isolated from a PRV-positive pig farm in Jilin, China [33]) was propagated in the PK-15 cells and confirmed via real-time polymerase chain reaction (real-time PCR). Infection experiments were performed in the Biosafety Level-2 (BLS-2) facility [32].

### 2.2. Plasmids

Recombinant plasmids were synthesized by Jinweizhi Biotechnology Co., Ltd. (Jiangsu, China). The PRV UL41 gene (GenBank: OP168821) was inserted into a pEGFP-N1 vector, resulting in the pEGFP-UL41 construct. The coding sequences (CDSs) of porcine-tripartite motif protein 21 (TRIM21, GenBank: NM_001163649) or human TRIM21 (GenBank: NM_003141) were, respectively, inserted into the pIRES-eGFP-N1 vector, generating pIRES-TRIM21 (porcine or human-derived).

The Hieff Mut Site-Directed Mutagenesis Kit (11003ES10, Yeason, Shanghai, China) was used to generate recombinant plasmids expressing mutants, including pIRES-TRIM21ΔRING (RING domain deleted at residues 16–54 aa) and pIRES-TRIM21ΔPRY/SPRY (SPRY/PRY domain deleted at 286–466 aa) of porcine-derived TRIM21, as well as pEGFP-UL41-F78A (F78A mutation of PRV UL41) with the indicated primers (Table S1). All constructs were confirmed by DNA sequencing.

### 2.3. Transfection

HEK293T and PK-15 cells were seeded to ~80% confluency and transfected with the indicated plasmids using EZ Cell Transfection Reagent (AC04L092, Shanghai Life-iLab Biotech Co., Ltd., Shanghai, China) according to the manufacturer's guidelines. After that, HEK293T cells were cultured in DMEM with 10% FBS, whereas PK-15 cells were cultured in DMEM with 5% FBS for 24–72 h before subsequent analysis.

### 2.4. Nucleotide Acid Extraction and Real-Time PCR

Nucleotide acid extraction and Real-time PCR were performed according to our previous protocol [32]. Briefly, viral genomic DNA was extracted using the BayBiopure Magnetic Viral DNA/RNA Kit in conjunction with the BayBioInvent K12 Automated Nucleic Acid Extractor (BayBio, Guangzhou, China), following the manufacturer's instructions.

Total RNA was isolated with TRNzol Universal Reagent (Epizyme, Shanghai, China). After that, cDNA was synthesized using the 5×All-In-One RT MasterMix (Abm, Canada), according to the manufacturer's guidelines.

Real-time PCR was performed on a Quantagene q225 real-time PCR system (Kubo Technology, China) using 2x SYBR Green qPCR Master Mix (Sellcek, Houston, TX, USA) with the indicated primers (Table S1). The PCR conditions were as follows: predenaturation at 95°C for 1 min; denaturation at 95°C for 15 s, annealing at 57°C for 15 s; and extension at 60°C for 30 s with a total of 40 cycles. The expression of target genes was normalized to the Ct values of the 18S rRNA gene. Relative mRNA expression was determined by comparing the results to the mock or Con group using the 2^-ΔΔCt^ method.

### 2.5. RNA Immunoprecipitation (RIP), RIP-Quantitative PCR (RIP-qPCR), and RIP-Seq

RIP was performed to evaluate the interaction between PRV UL41 and host mRNA. Briefly, cells (HEK293T and PK-15) at 80% confluency in 10 cm dishes were transfected with pEGFP-UL41 or pEGFP-UL41-F78A for 48 h. Transfection for 48 h was selected based on preliminary experiments showing peak UL41 expression at this time point (data not shown). Then, cells were lysed in lysis buffer for Western blotting and immunoprecipitation (WanleiBio, Shenyang, China) supplemented with protease inhibitors (Sellcek, Houston, TX, USA) and RNase inhibitor (E125, Novoprotein, Jiangsu, China) for 15 min on ice. The lysates were subsequently centrifuged at 12,000 rpm for 30 min. Proteins were incubated overnight at 4°C with anti-GFP antibody (rabbit, 1:100, HuaBio, Zhejiang, China). According to the manufacturer's protocol, the UL41-RNA complex was immunoprecipitated using protein A/G-magnetic beads (20 μL beads/10 μg antibody, Beyotime, Shanghai, China). The beads bound to the immunocomplexes were digested with protease K and the immunoprecipitated complexes were washed six times with RIP wash buffer (containing 150 mM KCl, 25 mM Tris pH 7.4, 5 mM EDTA, 0.5 mM DTT, 1 U/μL RNase inhibitor, and 1×protease inhibitor in ddH_2_O), followed by incubating with 150 uL proteinase K Buffer (1% SDS, 1.2 mg/mL proteinase K in RIP wash buffer) at 55°C for 30 min. The UL41-bound RNAs were eluted from the magnetic beads using Trizol reagent (Epizyme, Shanghai, China) and isolated according to the manufacturer's instructions.

For RIP-Seq analysis, the UL41-bound RNAs were processed using Trizol reagent for RNA extraction and analyzed by Personalbio (Shanghai, China). The input group was utilized as the Con.

Furthermore, the eluted UL41-GFP-bound RNAs were reverse-transcribed using the 5× All-In-One RT MasterMix (Abm, Canada), following the manufacturer's guidelines. The cDNA was amplified via real-time PCR, as described in Section 2.4. The RNA immunoprecipitated by PRV UL41 was normalized to the input, and rabbit IgG (1:100, Beyotime, Shanghai, China) was used as the isotype Con antibody. The fold of mRNA enrichment was calculated as (Ct [RIP]-Ct [input]) − (Ct [IgG]-Ct [input]). RIP-qPCR primers are listed in Table S1.

### 2.6. RNA Electrophoretic Mobility Shift Assay (EMSA)

According to the manufacturer's protocol, EMSA was conducted using an EMSA Kit (E33075, Thermo Scientific, Waltham, MA, USA). Briefly, single-strand RNA probes (Table S2), which correspond to KUUUCY and CSDGGA of the human Tyk2, STAT2, and IRF9 mRNAs (5′ and 3′-untranslated regions [UTRs]), were synthesized by Jinweizhi Biotechnology Co., Ltd. (Jiangsu, China). Purified UL41 protein (2 μM, Figure S1) was incubated with RNA probes (2 μM) in binding buffer for 40 min at 25°C. The UL41-RNA complexes were resolved on nondenaturing 10% polyacrylamide precast gels (AP15L714, Shanghai Life-iLab Biotech Co., Ltd., Shanghai, China). The gel was stained with 1×SYBR Green EMSA staining solution and visualized using 302 nm UV transillumination.

### 2.7. Coimmunoprecipitation (Co-IP) and Liquid Chromatography–Tandem Mass Spectrometry (LC–MS/MS)

Co-IP and LC–MS/MS were performed according to our previous protocol [32]. Briefly, cells were grown to 80% confluency in 10 cm culture dishes and transfected with pEGFP-UL41 or pEGFP-UL41-F78A for 48 h. Cells were lysed in a lysis buffer for Western and IP (WanleiBio, Shenyang, China) supplemented with protease inhibitors (Sellcek, Houston, TX, USA) for 15–20 min on ice, followed by centrifugation at 12,000 rpm for 20–30 min. Subsequently, 1 mg total protein was incubated with anti-GFP antibody (rabbit, 1:100, HuaBio, Zhejiang, China) overnight at 4°C. Following the manufacturer's protocol, immunoprecipitation was performed using protein A/G-magnetic beads (20 μL, Beyotime, Shanghai, China). The immunoprecipitated complexes were washed using acidic elution (0.1 M glycine-HCl, pH 3.0) and then neutralized with neutralization buffer (1 M tris-HCl, pH 7.4). As a Con, rabbit-IgG (1:100, Beyotime, Shanghai, China) was used as the specific antibody. The isolated immunoprecipitants were analyzed via LC–MS/MS by Personalbio (Shanghai, China) or Western blot. Nonspecific proteins from the rabbit-IgG group were subtracted from the dataset.

### 2.8. Western Blot

Western blot was conducted as previously described [32]. Briefly, cells were harvested and lysed in a cell lysis buffer for Western and IP (WanleiBio, Shenyang, China), supplemented with protease inhibitors (Sellcek, Houston, TX, USA). Protein was resolved by 10% SDS–PAGE and transferred onto a 0.45 μm PVDF membrane (Millipore, Billerica, MA, USA). Subsequently, the membrane was subjected to blocking with Protein Free Rapid Blocking Buffer (1×, PS108P, Epizyme, Shanghai, China) for 20 min at 22–25°C, followed by incubation with the indicated primary antibodies overnight at 4°C. The primary antibodies anti-GFP (rabbit, 1:2000), anti-TRIM21 (rabbit, 1:5000), anti-IFITM3 (rabbit, 1:2000), and anti-β-actin (mouse, 1:10,000) were purchased from Proteintech (Wuhan, China). Antibodies anti-eIF4E (rabbit, 1:1000), anti-PABPC1 (rabbit, 1:1000), anti-26S proteasome (rabbit, 1:1000), and anti-ISG15 antibody (rabbit, 1:1000) were from Baijia (Jiangsu, China). Anti-His antibody (mouse, 1:2000) was from ABclonal (Wuhan, China). Antibodies anti-Tyk2 (rabbit, 1:1000) and anti-STAT1 (rabbit, 1:1000) were from Affinity (Jiangsu, China). Antibodies anti-ubiquitin (mouse, 1:2000) and anti-K48 ubiquitin antibody (rabbit, 1:1000) were from Abmart (Shanghai, China). Antibodies anti-IRF3 (rabbit, 1:1000), anti-STAT2 (rabbit, 1:1000), anti-SOCS1 (rabbit, 1:500), anti-IFNα (rabbit, 1:750), and anti-IFNβ antibody (rabbit, 1:1000) were from WanleiBio (Shenyang, China). The anti-STING antibody (rabbit, 1:1000) was from Bioss (Beijing, China). The anti-IRF9 antibody (rabbit, 1:1000) was purchased from Cusabio (Wuhan, China).

After that, the membrane was incubated with HRP-conjugated secondary antibody (1:5000, Abmart, Shanghai, China) or HRP-conjugated Goat anti-mouse or rabbit IgG (H + L) (1:10,000, Abbkine, Wuhan, China) for 2 h at 22–25°C. Where necessary, the membrane was treated with a stripping solution (Applygen, Beijing, China) at 22–25°C for 30 min, washed with TBST (20 mM Tris and 150 mM NaCl, 0.2% Tween-20, pH 7.4), and reprobed with the antibody of interest. Protein bands were visualized using an ECL kit (Abbkine, Wuhan, China) and analyzed using a bioanalytical imaging system. The average expression level of the target protein in each group was normalized to β-actin and shown below each lane. The protein quantity in the untransfected group was set as the reference (designated as 1), and the values of the other groups are the ratios of the untransfected group.

### 2.9. Confocal Microscopic Analysis

Confocal microscopic analysis was performed according to our previous protocol [32]. Briefly, after transfection with pEGFP-UL41 for 48 h, PK-15 cells were seeded at a density of 10^5^ per well on slides in 24-well plates, and the cells were fixed in 80% acetone at −80°C overnight, washed with PBS twice (5 min each), and blocked with 3% bovine serum albumin (BSA) for 2 h at 22–25°C. Cells were incubated with anti-eIF4E (1:100), anti-PABPC1 (1:100), or anti-TRIM21 (1:500) at 22–25°C for 2 h, respectively. After washing with PBS, the cells were incubated in a dark box with anti-rabbit IgG-Cy3 (1:500, Abbkine, Wuhan, China) at 22–25°C for 2 h. Then, the cell nucleus was stained with DAPI (2 μg/mL, Boster, Wuhan, China) for 10 min at 22–25°C in a dark box. Finally, the slides were mounted with antifade mounting medium (Abbkine, Wuhan, China) and examined using the Nikon A1 confocal microscope.

### 2.10. Sequence Alignment, Domain Analysis, and Mutation Analysis

The information on UL41 proteins of herpesviruses is listed in Table S3. Sequence alignment, domain analysis, and mutation analysis were performed according to our previous protocol [32]. Briefly, the UL41 (PRV JL-21 strain) was used as the reference. The multiple alignments were conducted by Mega 11.0.13. TRIM21 domains were analyzed using Pfam (http://pfam.xfam.org/, accessed April 20, 2025) [34]. The mutation analysis of amino acids of UL41 was performed on the predicted structures using Missense3D [35] (http://missense3d.bc.ic.ac.uk/~missense3d/, accessed on December 31, 2024).

### 2.11. Three-Dimensional (3D) Modeling and Protein–Protein Docking

3D modeling and protein–protein docking were performed according to our previously established protocol [32]. Briefly, the 3D models of PRV UL41 (GenBank: WEU66586), human TRIM21 (GenBank: NP_003132), and porcine TRIM21 (GenBank: NP_001157121) were constructed using iDrug (Tencent AI Lab, https://drug.ai.tencent.com/). The structural complex was evaluated using the HDOCK server (http://hdock.phys.hust.edu.cn/). Images were generated by the Pymol Molecular Graphics System (version 2.0, Schrödinger, LLC).

### 2.12. Statistical Analysis

Statistical analyses were performed using GraphPad Prism 9.0 software, following the protocol described previously [32]. When comparing Con or mock groups with experimental groups, one-way or two-way analysis of variance (ANOVA) was employed, followed by post hoc tests (e.g., Dunnett's or Bonferroni's method) to adjust *p*-values. For comparisons between two groups, an unpaired *t*-test was used. Data from three independent experiments are presented as mean ± standard deviation (SD). *⁣*^*∗*^, *p*  < 0.05; *⁣*^*∗∗*^, *p*  < 0.01; *⁣*^*∗∗∗*^, *p*  < 0.001.

## 3. Results

### 3.1. PRV Modulates IFN Downstream Signaling via Suppression of the JAK/STAT Pathway

Prior investigations have shown that human cells are susceptible to PRV infection [32, 36]. PRV strains adapted to human cells can infect multiple cell lines, encompassing other human cell types, as well as PK-15 (porcine kidney cell) and Vero (monkey kidney cell) cells, culminating in the manifestation of cytopathic effects. Furthermore, the expression of antiviral factors was inhibited within PRV-infected PK-15 cells, particularly in those coinfected with PCV2 [37, 38]. These findings suggest that PRV activates immune evasion strategies to facilitate its replication and spread within the host upon entering host cells.

**Figure 1:**
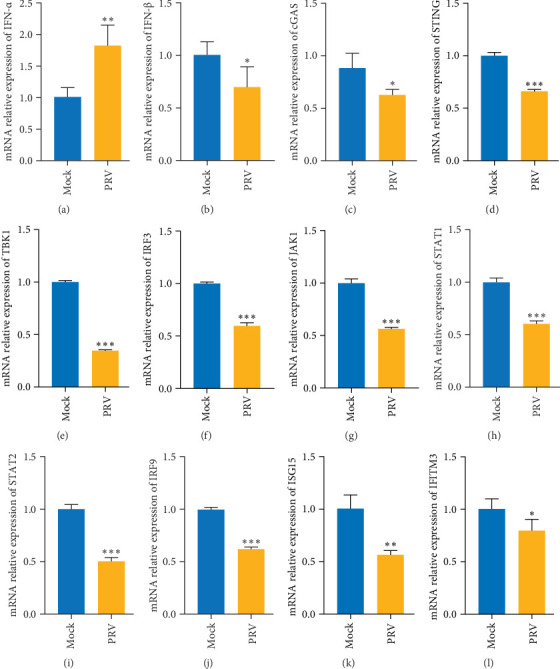
PRV suppresses IFN pathways in HEK293 cells. The mRNA of IFN-α (A), IFN-β (B), cGAS (C), STING (D), TBK1 (E), IRF3 (F), JAK1 (G), STAT1 (H), STAT2 (I), IRF9 (J), ISG15 (K), and IFITM3 (L) were detected using real-time PCR (*n* = 3 independent experiments). Mock, uninfected group; PRV, PRV infected group. *⁣*^*∗*^*p* < 0.05; *⁣*^*∗∗*^*p* < 0.01; *⁣*^*∗∗∗*^*p* < 0.001.

To investigate the impact of PRV on IFN responses in human cells, HEK293 cells were infected with PRV for 36 h. Subsequently, the expression levels of IFN-related genes were evaluated through real-time PCR. As illustrated in Figure 1, a substantial upregulation in the expression of IFN-α was discerned in HEK293 cells at 36 h post-infection (hpi) (Figure 1A). Conversely, the expression of IFN-β was attenuated within the PRV-infected cells (Figure 1B). IFN-α mRNA levels increased ~1.8-fold in PRV-infected cells, while IFN-β mRNA decreased by ~40%.

Moreover, the expression levels of pivotal constituents within the cGAS-STING pathway, namely cGAS, STING, TBK1, and IRF3, were also markedly diminished (Figure 1). In a parallel manner, the mRNA levels of essential genes within the JAK/STAT pathway, such as JAK1, STAT1, STAT2, and IRF9, were likewise conspicuously reduced (Figure 1). Additionally, a significant downturn in the expression of antiviral factors (ISG15 and IFITM3) was also detected (Figure 1K,L). These findings imply that PRV can modulate the downstream signaling of IFN by repressing the JAK/STAT pathway at the transcriptional level. This, in turn, leads to a decreased expression of the antiviral factors ISG15 and IFITM3, thereby potentially facilitating the evasion of the host immune response and creating a more favorable environment for viral replication and persistence.

### 3.2. PRV UL41 Orchestrates the JAK/STAT Pathway via Binding to the UTRs of Target mRNAs

**Figure 2:**
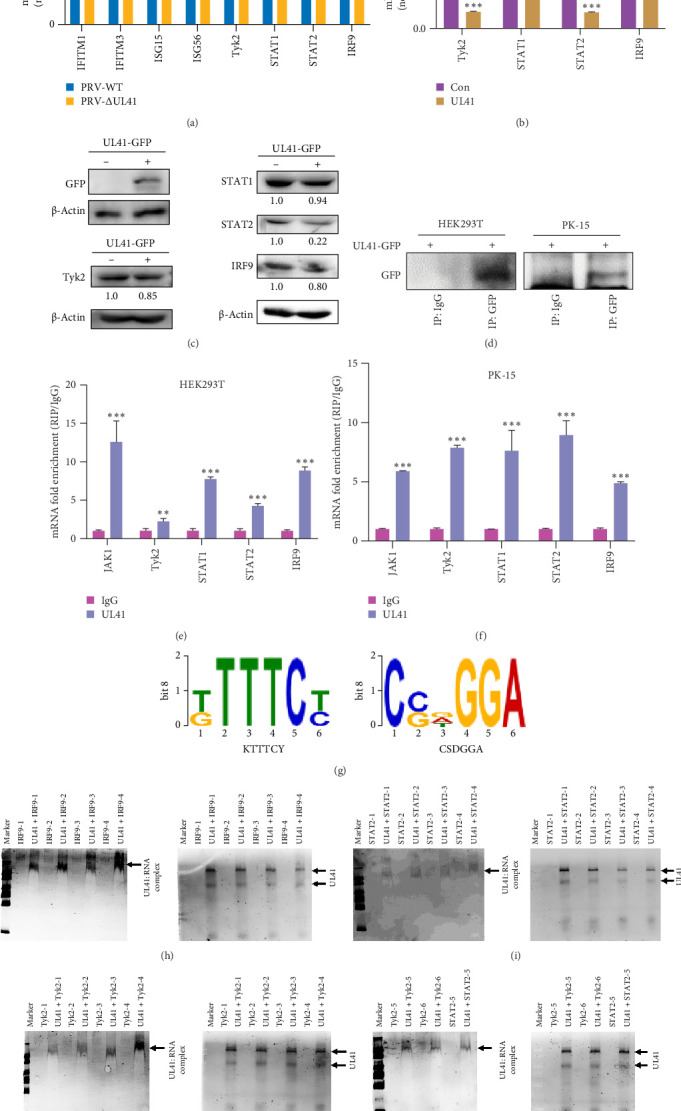
PRV UL41 could bind to the UTR region of the target mRNA of the JAK/STAT pathway. (A) PK-15 cells were infected with PRV-WT and PRV-ΔUL41 for 36 h. The levels of IFITM1, IFITM3, ISG15, ISG56, Tyk2, STAT1, STAT2, and IRF9 were determined by real-time PCR. (B,C) HEK293T cells were transfected with pEGFP-UL41 for 48 h. The levels of Tyk2, STAT1, STAT2, and IRF9 were determined by real-time PCR (A) and western blot (B). Control (Con) and minus (−): untransfected; plus (+): transfected with the pEGFP-UL41 plasmid. (D–F) HEK293T and PK-15 cells were transfected with pEGFP-UL41 for 48 h, respectively, followed by RIP using the anti-GFP antibody. Then, the RIP complex was evaluated via western blot (D) or RT-qPCR (E,F). (G) Consensus motifs bound to PRV UL41 identified by RIP-seq in HEK293T cells. (H–K) RNA EMSA shows the binding results of probes with motifs as indicated in (G) to PRV UL41. PRV UL41 protein (2 μM) was incubated with RNA probes (2 μM) of IRF9 (H), STAT2 (I,K), and Tyk2 (J,K) in the binding buffer for 40 min at 25°C, followed by the EMSA assay. Marker, DNA Marker 2000. *⁣*^*∗*^*p* < 0.05; *⁣*^*∗∗*^*p* < 0.01; *⁣*^*∗∗∗*^*p* < 0.001.

Herpesvirus UL41 proteins have been shown to directly or indirectly impact the JAK/STAT pathway, thereby modulating immune responses [39, 40]. Therefore, this study focuses on PRV UL41 to elucidate its role in modulating the JAK/STAT pathway and uncover the intricate molecular mechanisms involved. PK-15 cells were infected with PRV-WT and PRV UL41 knockout strain (PRV-ΔUL41, Figure S2) for 36 h. The levels of IFITM1, IFITM3, ISG15, ISG56, Tyk2, STAT1, STAT2, and IRF9 mRNAs were examined. As shown in Figure 2A, the levels of IFITM1, IFITM3, ISG56, and STAT2 mRNAs were upregulated significantly in the UL41-knockout group compared with those of the wild-type group. Furthermore, PRV UL41 was overexpressed in HEK293T cells for 48 h, and the expression profiles of JAK/STAT-related genes were evaluated. The results demonstrated that, in contrast to Con cells, the mRNA and protein abundances of Tyk2, STAT1, STAT2, and IRF9 were decreased in cells overexpressing UL41 (Figure 2B,C). Given that Tyk2 and STAT2 are integral proteins in the specific transduction of IFN signals within the JAK/STAT pathway [41], these data suggest that PRV UL41 may suppress IFN-I signaling by targeting the JAK/STAT pathway, consequently influencing the expression of antiviral factors and curtailing the intracellular immune response.

To delineate the target mRNA sequences of PRV UL41 responsible for its inhibitory effect on the JAK/STAT pathway, HEK293T and PK-15 cells were transfected with a PRV UL41 expression plasmid for 48 h, followed by RIP or RIP-qPCR assays. As depicted in Figure 2, the PRV UL41 protein (Figure 2D), as well as the mRNAs of JAK1, Tyk2, STAT1, STAT2, and IRF9 (Figure 2E,F), were detectable in the RIP mixture, signifying that the mRNAs of JAK1, Tyk2, STAT1, STAT2, and IRF9 are among the targets of PRV UL41 during infection.

Subsequently, the RIP mixture was subjected to RIP-seq analysis to elucidate the UL41-interacting motifs within the mRNAs. As anticipated, conserved sequences KUUUCY and CSDGGA, which bind to the UL41 protein, were identified in the UTRs of the Tyk2, STAT2, and IRF9 mRNAs (Figure 2G). Thereafter, RNA EMSA was performed to validate the interaction between PRV UL41 and the mRNAs encoding Tyk2, STAT2, and IRF9, using probes designed based on conserved sequences within these mRNAs (Table S4). As shown in Figure 2, specific bands (UL41:RNA complex) were detected in the UL41-RNA probe groups, indicating that all RNA probes corresponding to the conserved sequences could interact with the UL41 protein. Therefore, the KUUUCY and CSDGGA sequences represent the critical motifs within the mRNAs of Tyk2, STAT2, and IRF9, key components of the JAK/STAT pathway. These motif-containing mRNAs can be specifically recognized and degraded by PRV UL41, ultimately leading to the downregulation of the host antiviral immune response during PRV infection.

### 3.3. Residue F78 of UL41 Constitutes a Pivotal Amino Acid in the Inhibition of the JAK/STAT Pathway by UL41

**Figure 3:**
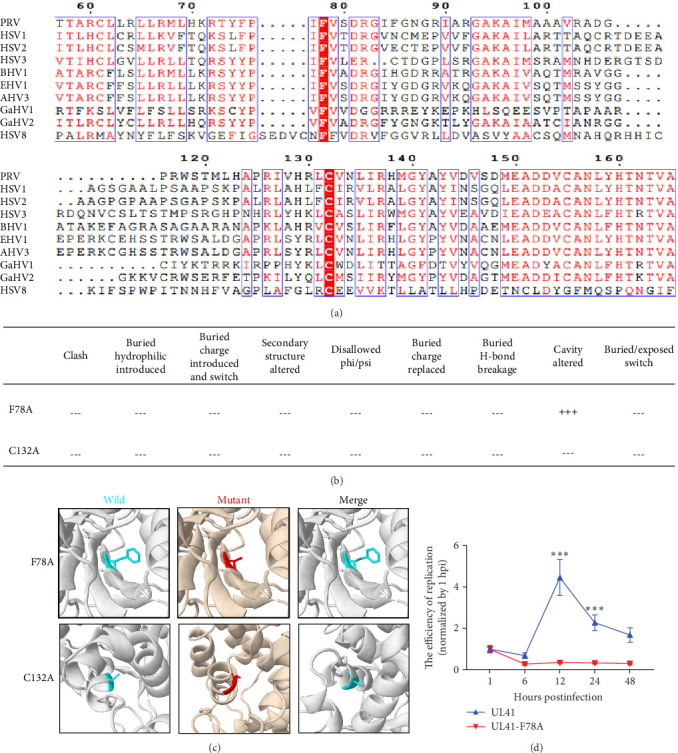
Conserved residues and mutation analysis of Herpesvirus UL41/vhs protein. (A) The conserved residues analysis of PRV UL41 compared with that of the HSV1, HSV2, HSV3, BHV1, EHV1, AHV3, GaHV1, GaHV2, HSV8. (B,C) Mutation analysis of PRV UL41 F78 and C132. (B) Structural comparisons with the wild-type protein and mutants. (C) The F78A and C132A mutations affect the architectural integrity of the PRV UL41 protein. “+” means difference, and “−” means no difference. (D) The effect of UL41 F78 on the proliferation of PRV. HEK293 cells were respectively transfected with pEGFP-UL41 and pEGFP-UL41-F78A for 36 h. Then, cells were infected with PRV, and the genomic copy number of PRV was determined within 1–48 hpi using real-time PCR. The replication efficiency for each time was calculated using the copy number at one hpi in each group as the baseline. *⁣*^*∗∗∗*^*p* < 0.001.

As previously reported, the UL41 protein exhibits high conservation within the Alphaherpesvirus subfamily [10, 11]. Through sequence comparisons among herpesviruses (Table S3), residues F78 (Phenylalanine, F) and C132 (cysteine, C) within the UL41 were identified as conserved residues (Figure 3A). Moreover, the 3D model of UL41 suggested that only the F78A mutation could substantially perturb the cavity-packing of UL41 (Figure 3B,C), intimating that the F78 within UL41 might serve as a critical functional residue across herpesvirus UL41 variants.

It has been reported that a dominant-negative mutant of a viral protein can restrict virus infection, such as HIV-1 and HSV-1 [42–44]. To comprehensively elucidate the role of phenylalanine at position 78 (F78) within PRV UL41, HEK293 cells were respectively transfected with pEGFP-UL41 and pEGFP-UL41-F78A (replacing F78 with alanine), followed by infection with PRV. As shown in Figure 3D, during infection, the replication kinetics of the PRV were substantially reduced in cells overexpressing UL41-F78A compared to those of the wild-type UL41 group. These findings provide evidence for the indispensable role of F78 in enabling UL41 to facilitate efficient PRV replication.

**Figure 4:**
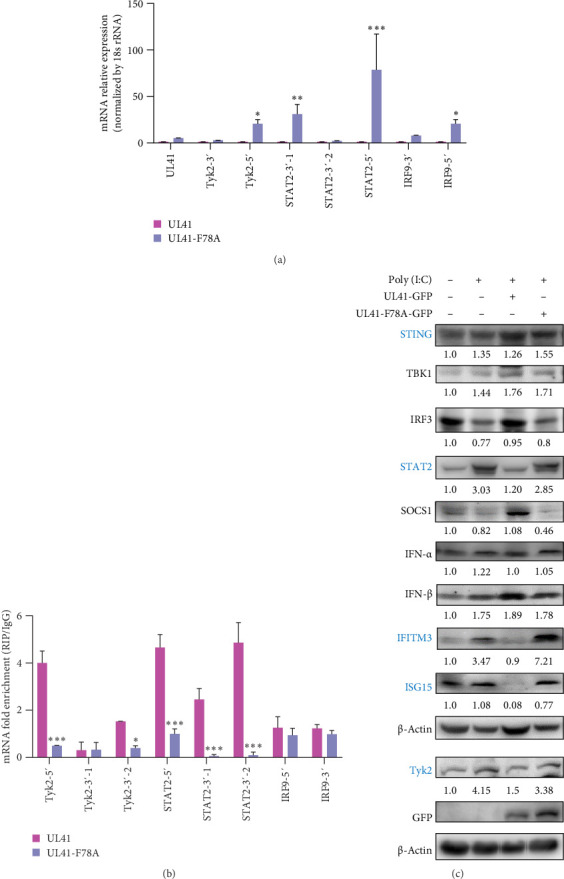
Evaluation of the F78A mutation of PRV UL41 on the JAK/STAT pathway. (A,B) HEK293T cells were transfected with pEGFP-UL41 or pEGFP-UL41-F78A for 48 h. The 3′ and 5′-UTR of Tyk2, STAT2, and IRF9 mRNAs were determined by RT-qPCR (A). The 3′ and 5′-UTR of Tyk2, STAT2, and IRF9 mRNAs bound to the PRV UL41 were detected via RIP using GFP–specific antibody, followed by RT-qPCR (B). (C) HEK293T cells were transfected with pEGFP-UL41 or pEGFP-UL41-F78A for 48 h, followed by treatment with poly (I:C) for 12 h, and evaluated via Western blot. Minus (−): untransfected or untreated with poly (I:C); plus (+): transfected with the indicated plasmid or treated with poly (I:C). *⁣*^*∗*^*p* < 0.05; *⁣*^*∗∗*^*p* < 0.01; *⁣*^*∗∗∗*^*p* < 0.001.

To further dissect the impact of F78 on the RNase activity of PRV UL41, the levels of 3′ and 5′ UTR in Tyk2, STAT2, and IRF9 mRNAs in cells transfected with pEGFP-UL41 and pEGFP-UL41-F78A were evaluated via RT-qPCR or RIP/RT-qPCR. The results showed that the mRNA levels of the 3′- and/or 5′-UTRs of Tyk2, STAT2, and IRF9 were markedly elevated in the UL41-F78A overexpressed group compared to the wild-type group (Figure 4A). Additionally, RIP results demonstrated that the wild-type UL41 predominantly interacts with the 3′- and 5′-UTR of Tyk2 and STAT2 mRNAs (motif sequences: KUUUCY and CSDGGA) (Figure 4B). In contrast, the F78A mutation in the UL41 decreased its binding to the 3′- and 5′-UTR of Tyk2 and STAT2, yet had no discernible effect on its binding to the IRF9 mRNA. These findings imply that the F78A mutation in PRV UL41 attenuates the RNase activity of UL41 towards target mRNAs, evidenced by higher levels of Tyk2 and STAT2 UTR mRNAs in UL41-F78A-overexpressing cells (Figure 4A), by impairing its binding to these targets, thereby mitigating the suppression of protein expression.

Furthermore, intracellular immune signaling was activated by poly(I:C), instigating an upsurge in the expression of STING, TBK1, IFN-α, IFN-β, and proteins within the downstream JAK/STAT pathway, such as Tyk2 and STAT2, as well as the expression of immune-related factors ISG15 and IFITM3 (Figure 4C). However, the overexpression of UL41 downregulated the expression of STING, STAT2, IFITM3, ISG15, and Tyk2 relative to the poly(I:C) group, signifying that PRV UL41 represses the activation of the JAK/STAT pathway stimulated by poly(I:C), consequently suppressing downstream IFN signaling and ultimately diminishing the levels of ISG15 and IFITM3. As anticipated, the UL41 F78A mutation attenuated the influence of UL41 on the regulation of the JAK/STAT-IFN pathway by poly(I:C), resulting in increased levels of STING, STAT2, IFITM3, ISG15, and Tyk2. These results further corroborate that residue F78 within the UL41 is a crucial amino acid for the UL41 to inhibit the JAK/STAT pathway.

### 3.4. F78 Represents a Critical Residue for the Interaction of UL41 With Target Proteins

Previous reports have indicated that the HSV vhs can bind to eIF4A and eIF4H and translocate the PABPC1 to the nucleus [18–21]. It is hypothesized that the ability of vhs to degrade host mRNAs redirects the host's translational machinery towards viral mRNAs, thereby promoting the translation of viral mRNAs and enhancing viral protein synthesis and replication. To ascertain whether PRV UL41 exhibits analogous characteristics, HEK293T cells were transfected with the pEGFP-UL41 for 48 h, followed by Co-IP and LC–MS/MS analyses to identify proteins involved in the recognition of UL41-mRNA. The LC–MS/MS results revealed that several translation-related proteins were detected within the Co-IP complex, with eIF4E and PABPC1 being the most prominent. Therefore, the interaction between PRV UL41 and eIF4E or PABPC1 was further evaluated.

**Figure 5:**
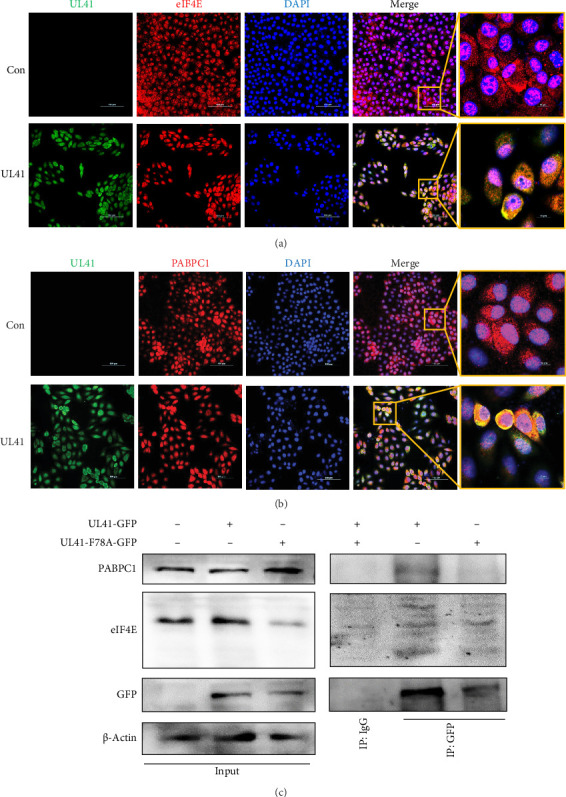
PRV UL41 can bind to eIF4E and PABPC1. (A,B) PK-15 cells were transfected with pEGFP-UL41 for 48 h, followed by colocalization of UL41 (GFP) and eIF4E (A) or PABPC1 (B) using anti-eIF4E/anti-PABPC1 antibodies (red). UL41 was fuzed with GFP (green), and the cell nucleus was stained with DAPI (blue). Scale bar, 100 μm (left 1–4 panel) and 10 μm (enlarged). (C) PK-15 cells were transfected with pEGFP-UL41 or pEGFP-UL41-F78A for 48 h, followed by Co-IP and western blot. Minus (−): untransfected; plus (+): transfected with the indicated plasmid.

As a result, the colocalization of PRV UL41 with eIF4E and PABPC1 was observed within the cells, spanning both the nucleus and cytoplasm (Figure 5A,B). The outcomes of Co-IP and Western blot assays provided conclusive evidence of the interaction between PRV UL41 and the cellular PABPC1 proteins, but its interaction with eIF4E is not apparent (Figure 5C). Significantly, the UL41-F78A mutant did not appreciably affect the binding of UL41 to eIF4E. However, it did lead to a reduction in its interaction with PABPC1. These findings suggest that the F78 residue within PRV UL41 may play a crucial role in its interaction with PABPC1, potentially facilitating its association with the UTR of target mRNAs. This residue thus emerges as a crucial determinant in the molecular interaction between UL41 and its binding partners.

### 3.5. The Antagonistic Interaction Between PRV UL41 and TRIM21

**Figure 6:**
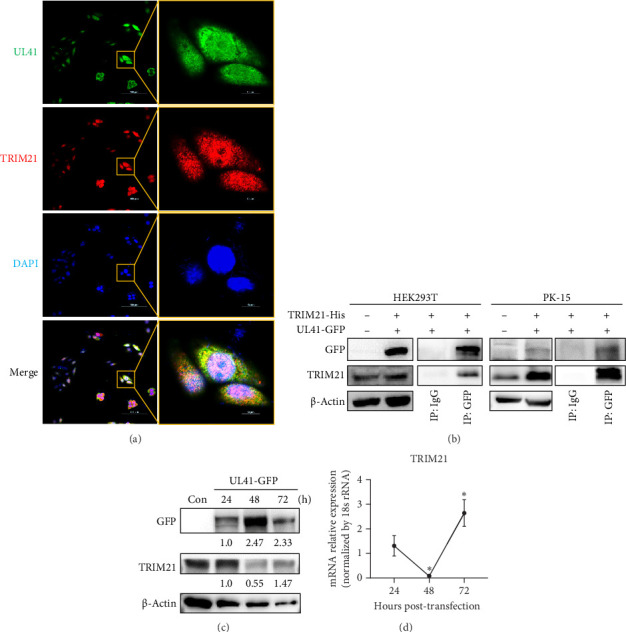
PRV UL41 interacted with cellular TRIM21. (A) PK-15 cells were transfected with pEGFP-UL41 for 48 h and examined by confocal microscopy using anti-TRIM21 antibody (red) and GFP (UL41, green). The cell nucleus was stained with DAPI (blue). Scale bar, 100 μm (left) and 10 μm (right). (B) HEK293T and PK-15 cells were transfected with pEGFP-UL41 and pIRES-TRIM21 (human or porcine-derived) for 48 h, followed by Co-IP with anti-GFP antibody or rabbit IgG and Western blot. (C,D) HEK293T was transfected with pEGFP-UL41 for 24–72 h, followed by western blot (C), and the expression levels of TRIM21 were evaluated by RT-qPCR (D). *⁣*^*∗*^*p* < 0.05.

Intriguingly, tripartite motif protein 21 (TRIM21), a well-established antiviral factor [45], was also detected among the cellular proteins involved in UL41-mRNA interactions through Co-IP and LC–MS/MS analyses. To elucidate the nature of the interaction between UL41 and TRIM21, PRV UL41 was overexpressed in PK-15 cells. As shown in Figure 6A, PRV UL41 and endogenous TRIM21 were colocalized within the cell, predominantly in the cytoplasm. This interaction was further confirmed by overexpressing PRV UL41 in both HEK293T and PK-15 cells, followed by Co-IP and Western blot assays. As expected, cellular TRIM21 could be effectively coimmunoprecipitated with PRV UL41 in both cell types (Figure 6B), confirming the binding affinity between PRV UL41 and cellular TRIM21, including human and porcine TRIM21.

Subsequently, upon overexpression of UL41 in HEK293T cells, the levels of UL41 peaked at 48 h post-transfection, followed by a slight decline at 72 h post-transfection (Figure 6C). In contrast, the protein and mRNA levels of endogenous TRIM21 decreased at 48 h post-transfection but then showed a marked increase after 72 h post-transfection (Figure 6C,D). This expression kinetics suggest an underlying antagonistic relationship between cellular TRIM21 and PRV UL41. Specifically, an increase in the UL41 protein was concomitant with a decrease in the TRIM21 protein. The amount of PRV UL41 within the coexpressed cells exhibited a dose-dependent reduction in response to TRIM21. These results firmly establish the antagonistic interplay between PRV UL41 and cellular TRIM21, which likely represents an important aspect of the host–pathogen dynamic, with implications for the balance between viral replication and the host's antiviral defenses.

### 3.6. TRIM21 Suppresses PRV UL41 Through the K48-Mediated Ubiquitin-Proteasome Pathway

**Figure 7:**
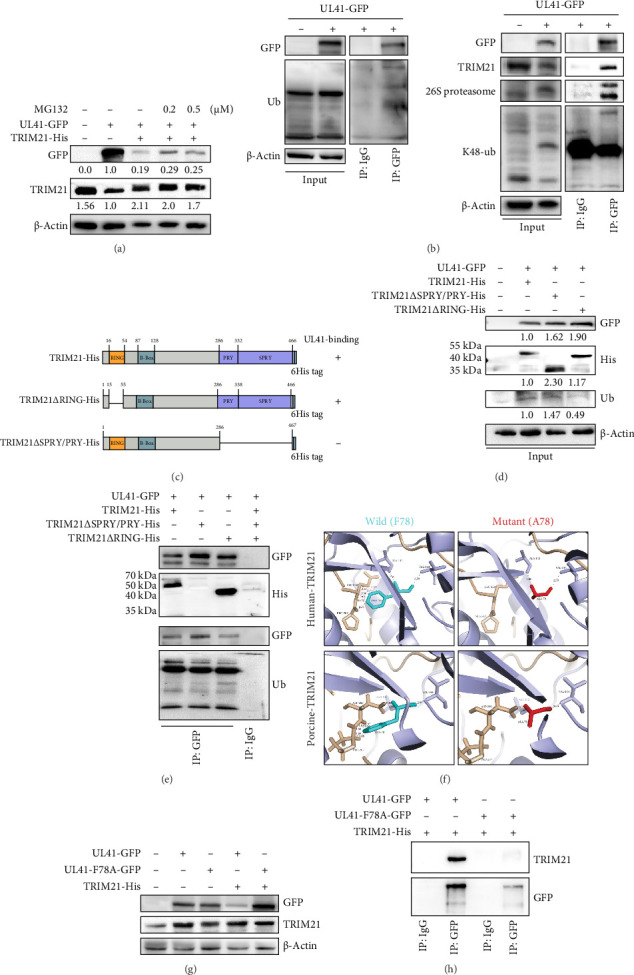
TRIM21 mediates the suppression of UL41 expression via the ubiquitin-proteasome pathway. (A) HEK293T cells were cotransfected with pEGFP-UL41 and pIRES-TRIM21 (porcine) plasmids for 36 h, followed by treatment with MG132 for 12 h and Western blot assay. (B) HEK293T cells were transfected with pEGFP-UL41 for 48 h, followed by Co-IP with anti-GFP antibody or rabbit IgG, and then subjected to Western blot analysis. (C) Schematic diagram of porcine-derived TRIM21 wild-type (TRIM21-His) or mutants with RING (TRIM21ΔRING-His) or PRY/SPRY (TRIM21ΔPRY/SPRY-His) domain deleted. (D,E) HEK293T cells were cotransfected with pEGFP-UL41 and TRIM21 expressing plasmids, including pIRES-TRIM21, pIRES-TRIM21ΔRING, or pIRES-TRIM21ΔPRY/SPRY for 48 h, followed by western blot (D) and Co-IP (E) with anti-GFP antibody or rabbit IgG. (F) Predicted interaction model between TRIM21 (human/porcine-derived) and PRV UL41. TRIM21, wheat; PRV UL41, light blue. The labels highlight the residues involved in its interaction. The black dashed line indicates hydrogen bonds within PRV UL41, while the red dashes represent hydrogen bonds between PRV UL41 and TRIM21. The root mean square deviation values were presented as numerical values. Residue F78 of UL41 was indicated by cyan, and mutated A78 was indicated by red. (G,H) HEK293T cells were cotransfected with pIRES-TRIM21 (derived from porcine) and pEGFP-UL41 or pEGFP-UL41-F78A for 48 h, followed by western blot (G) and Co-IP (H). Minus (−): untransfected; plus (+): transfected with the indicated plasmid.

TRIM21, acting as an E3 ubiquitin ligase, is known to trigger the degradation of target proteins via the ubiquitin-proteasome pathway [45]. To ascertain whether TRIM21 exerts its inhibitory effect on PRV UL41 through this pathway, HEK293T cells were transfected with plasmids encoding PRV UL41 and TRIM21 with or without treatment of MG132 at an appropriate concentration (Figure S3) during transfection. As depicted in Figure 7A, the overexpression of PRV UL41 markedly attenuated the level of endogenous TRIM21. Conversely, when TRIM21 and UL41 were coexpressed within the cells, the level of TRIM21 rose, while that of UL41 decreased conspicuously, further substantiating the reciprocal inhibitory relationship between TRIM21 and PRV UL41. Notably, treatment with MG132 led to a pronounced increase in the level of PRV UL41 in TRIM21-overexpressing cells compared to the untreated Cons (Figure 7A). This observation strongly indicates that TRIM21 inhibits PRV UL41 through the ubiquitin-proteasome pathway. Moreover, PRV UL41 engages in interactions with ubiquitin, TRIM21, the 26S proteasome, and K48-ubiquitin (Figure 7B). These findings suggest that the level of PRV UL41 is modulated by the TRIM21-mediated K48-ubiquitin-proteasome pathway, which represents a crucial regulatory mechanism in the host–virus interaction and may offer potential targets for antiviral intervention strategies.

The RING and SPRY/PRY domains constitute two essential structural components of TRIM21 [45]. To assess the functions of the SPRY/PRY and RING domains in the degradation of PRV UL41 induced by TRIM21, truncated mutants of TRIM21 were engineered, with either the SPRY/PRY domain (TRIM21ΔSPRY/PRY-His) or the RING domain (TRIM21ΔRING-His) deleted (Figure 7C). Upon coexpressing PRV UL41 with the wild-type TRIM21 or its truncated counterparts lacking the SPRY/PRY or RING domains in HEK293T cells, the levels of both TRIM21 and UL41 were substantially elevated in the TRIM21 domain-deleted groups relative to the wild-type group (Figure 7D). Furthermore, ubiquitin levels increased in the SPRY/PRY deleted group but decreased in the RING deleted group compared with the wild-type group. These findings suggest that the SPRY/PRY and RING domains of TRIM21 participate in the interaction and ubiquitination-mediated degradation of UL41. Moreover, both the wild-type and RING domain-deleted forms of TRIM21 could be effectively immunoprecipitated by UL41, whereas the SPRY/PRY domain-deleted variant could not (Figure 7E), further validating the crucial role of the SPRY/PRY domain in the binding of TRIM21 to PRV UL41. These results suggest that TRIM21 facilitates the ubiquitination and subsequent degradation of UL41 through its interaction mediated by the SPRY/PRY domain, representing a key regulatory mechanism in the host–virus interaction dynamics.

Furthermore, the predicted interaction model between TRIM21 and PRV UL41 indicated that the UL41-F78A mutation would diminish the interaction between TRIM21 and UL41 (Figure 7F). The results of Western blot and Co-IP assays further corroborated that the F78A mutant of PRV UL41 displayed a reduced binding affinity for TRIM21 compared to the wild-type UL41 group (Figure 7G,H). These results provide additional evidence that F78 is also a critical residue in the interaction between PRV UL41 and TRIM21, highlighting its importance in the intricate network of host–virus interactions and potentially serving as a target for modulating viral pathogenesis and host immune responses.

### 3.7. TRIM21 Regulates the JAK/STAT Pathway Through Its Interaction With PRV UL41

**Figure 8:**
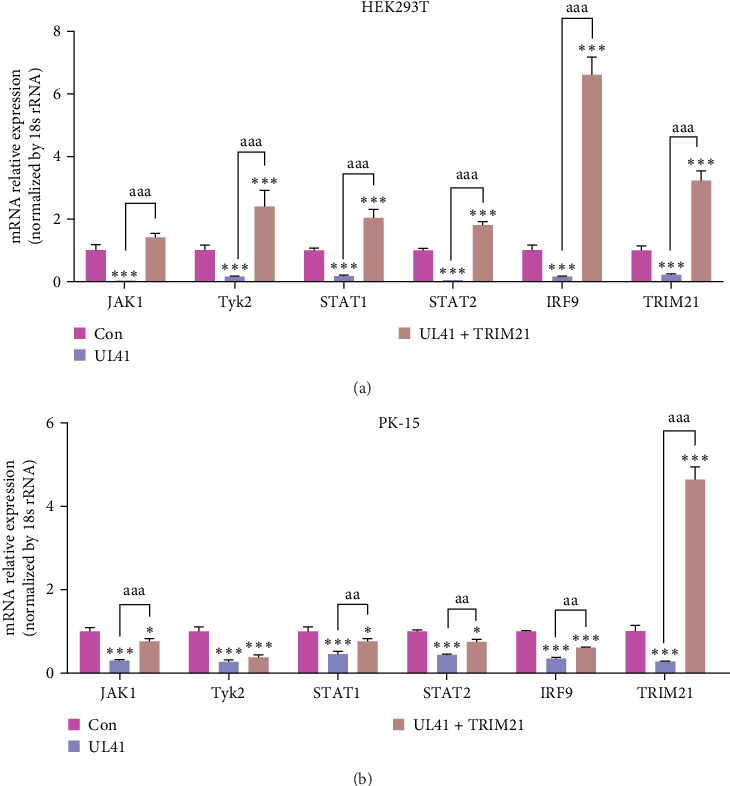
TRIM21 can counteract the effects of JAK/STAT signaling disrupted by UL41. HEK293T (A) and PK-15 (B) cells were transfected with pEGFP-UL41 or cotransfected with pEGFP-UL41 and pIRES-TRIM21 for 48 h. The relative levels of JAK1, Tyk2, STAT1, STAT2, IRF9, and TRIM21 mRNAs were detected by RT-qPCR. Control (Con): uninfected group. Compared to the Con group, *⁣*^*∗*^, *⁣*^*∗∗*^, and *⁣*^*∗∗∗*^ correspond to *p*-values of <0.05, <0.01, and <0.001, respectively. For the comparison between the transfected groups (UL41, UL41 + TRIM21), the designations a, aa, and aaa correspond to *p*-values of <0.05, <0.01, and <0.001, respectively.

Ultimately, the interplay between PRV UL41 and TRIM21 in the JAK/STAT pathway was investigated by overexpressing PRV UL41 and TRIM21 in both PK-15 and HEK293T cells. As illustrated in Figure 8, the mRNAs of cellular JAK1, Tyk2, STAT1, STAT2, IRF9, and TRIM21 were markedly diminished in the UL41-overexpressing cells compared to the Con group. However, when the cells were coexpressed with PRV UL41 and TRIM21, the levels of mRNAs for JAK1, STAT1, STAT2, IRF9, and TRIM21 were enhanced relative to those in the UL41 group. These results further validate that the interaction between PRV UL41 and TRIM21 modulates the JAK/STAT pathway. Specifically, PRV UL41 acts to suppress the activation of the JAK/STAT pathway, while TRIM21 counteracts this inhibition by degrading PRV UL41 via the ubiquitin-proteasome pathway, restoring the expression of JAK1, STAT1/2, and IRF9 mRNAs in both HEK293T and PK-15 cells (Figure 8). This dynamic interaction between the virus and the host protein represents a critical regulatory mechanism in the host–pathogen interface, with implications for understanding and potentially manipulating the balance between viral replication and the host immune response.

## 4. Discussion

Our prior investigations revealed that PRV can infect human cells, and we identified vimentin (VIM) as a universal receptor that facilitates PRV infection in both human and porcine cells [32, 36]. In the current study, we expanded our research scope by using PRV to infect HEK293 and PK-15 cells, aiming to comprehensively elucidate the infection mechanism of PRV within these cell types. Upon entry into the host cell, the viral double-stranded DNA of PRV promptly activates the cGAS sensor, thereby instigating a downstream signaling cascade that culminates in the activation of the signaling pathway comprising STING, TBK1, IRF3, and IFN-β [46–48]. As illustrated in Figure 1, PRV infection significantly suppresses the cGAS-STING-TBK-IRF3-IFN-β signaling at the mRNA level. This phenomenon unequivocally demonstrates that PRV can elude the antiviral immune response mediated by this signaling pathway. Ultimately, this results in a substantial attenuation of the antiviral effector factors ISG15 and IFITM3 expression. Collectively, these findings suggest that PRV strategically targets the production of the IFN-β pathway to achieve immune evasion.

Previously, numerous reports have shed light on PRV's diverse immune evasion strategies. For instance, PRV US3 [49] and UL13 [50] can precisely target IRF3, and PRV UL24 [51] regulates IRF7, suppressing cGAS-STING-mediated IFN-β activation. Additionally, PRV UL42, US3, and UL50 have been shown to inhibit the JAK/STAT pathway, thereby preventing the production of antiviral ISGs [52]. In the present study, however, we have uncovered the direct inhibitory role of the PRV UL41 protein on the JAK/STAT pathway. Within the intricate JAK/STAT signaling network, Tyk2, STAT2, and IRF9 play pivotal roles in relaying IFN-α/β signals [41]. Concurrently, SOCS1 can inhibit the phosphorylation of JAK1/Tyk2, thereby tempering IFN signal transduction [53]. Through RIP and RIP-seq analyses, we discovered that PRV UL41 can accurately recognize and bind to the sequences corresponding to KUUUCY and CSDGGA in the mRNAs of Tyk2, STAT2, and/or IRF9 (Figures 2 and 4). This interaction triggers mRNA degradation, thereby stymying IFN signaling and fortifying PRV's immune evasion.

The UL41 protein exhibits high conservation within the *A*lphaherpesvirus subfamily [10, 11]. It plays a crucial role in viral infection, causing mRNA degradation to halt host protein synthesis while protecting viral transcripts [10, 12–17]. UL41 also regulates viral gene expression and interacts with host factors, crucial for viral replication and immune evasion [54, 55]. Moreover, eIF4E, as a constituent of the eIF4F complex, can bind to the m^7^G cap structure of mRNA [56]. Meanwhile, PABPC1 safeguards mRNA from degradation by associating with its 3′ terminus and stimulates the assembly of eIF4F and the subsequent recruitment of ribosomal subunits, thereby potentiating protein synthesis [57]. During HSV1 infection, however, the UL41 protein interacts with eIF4F and PABPC1, rendering mRNA unstable and impeding the translation process [18, 19, 28, 58, 59]. In the present study, it was revealed that the PRV UL41 protein can also engage in reciprocal interactions with PABPC1 but may have no obvious interaction with eIF4E (Figure 5). The interaction between PRV UL41 and PABPC1 may enhance the UL41 binding to the motifs (KUUUCY and CSDGGA) within target mRNAs, culminating in mRNA cleavage and the suppression of the ensuing translation process. Additionally, the UL41 protein might interfere with the functions of translation initiation factors by binding to these factors, thereby repressing the translation process and gene expression within host cells. These findings further accentuate the pivotal role of the PRV UL41 in viral immune evasion. It achieves this by recognizing and binding to specific structures within host mRNA, such as regions abundant in AU-rich elements, and mediating mRNA degradation, which subsequently inhibits the IFN signaling and the antiviral responses of host cells. In addition, the functional consequences of UL41-PABPC1 from different herpesviruses and their permissive cells need to be evaluated to illustrate the mechanisms involved in virus infection, pathogenesis, and immune escape.

Previous research has illuminated the antiviral potency of TRIM21 in the context of various viral infections [45, 60]. This study detected an interaction between the host TRIM21 and PRV UL41 in HEK293T and PK-15 cells (Figure 6A,B). Further validation identified an antagonistic interplay between PRV UL41 and TRIM21 (Figure 6C,D). In HEK293T cells, upon overexpression of UL41, the protein abundance of TRIM21 decreased at 48 h post-transfection but then gradually increased. Similarly, when the expression level of TRIM21 was elevated, the quantity of UL41 protein was significantly reduced. These results suggest that the overexpression of TRIM21 may trigger specific intracellular responses, leading to a reduction in UL41 protein content and thereby alleviating the inhibitory effect on TRIM21. Hence, it is hypothesized that, as an E3 ubiquitin ligase, TRIM21 could mediate the degradation of PRV UL41 via the ubiquitination pathway, thereby restoring the antiviral responses of host cells. As expected, it was observed that TRIM21 induces the degradation of UL41 through the K48-dependent ubiquitin-proteasome pathway (Figure 7). Subsequent in-depth analyses of the pivotal domains showed that both the PRY/SPRY and RING domains of TRIM21 participate in the degradation of PRV UL41. When the PRY/SPRY domain was deleted, the binding between PRV UL41 and TRIM21 was abolished, resulting in a significant reduction in ubiquitin conjugated to UL41 and an increase in UL41 within the cells. Therefore, the SPRY/PRY domain of TRIM21 plays a crucial role in the binding to PRV UL41. Construction of genetically modified animal models (such as TRIM21 knockout or overexpression mice) is in progress to further investigate the TRIM21-UL41 interaction in virus infection and its pathogenesis under in vivo conditions.

Moreover, amino acid sequence alignments of UL41/vhs proteins across diverse herpesviruses were conducted (Figure 3). Intriguingly, the 78th amino acid (Phe, F) within PRV UL41 was identified as a conserved and functionally critical site among UL41/vhs proteins in herpesviruses. Upon mutating F78–A78, a discernible attenuation in PRV UL41's capacity to bind and cleave cellular mRNA was observed in cells (Figure 4). Concurrently, its binding affinity to PABPC1 proteins was significantly decreased (Figure 5). Besides, PRV UL41 can effectively interact with TRIM21 in cells. In contrast, the F78 of PRV UL41 was identified as one of the crucial binding sites for TRIM21 and UL41 (Figure 7). These findings underscore the pivotal role of the F78 in determining the functionality of PRV UL41 and potentially other herpesviruses. It could serve as a key functional residue in other herpesvirus UL41/vhs proteins, thus proffering valuable insights into the molecular underpinnings of the UL41 protein.

**Figure 9:**
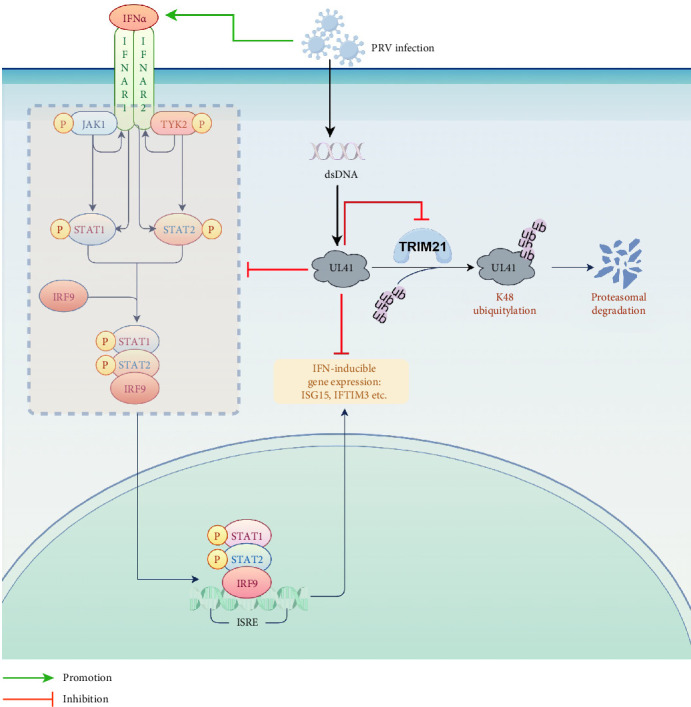
Schematic illustration of the immune escape of PRV induced by viral UL41. PRV UL41 inhibits the IFN-β signaling cascade by degrading the mRNA of the proteins in the JAK/STAT pathway, especially Tyk2 and STAT2. Additionally, PRV UL41 can inhibit the expression of the TRIM21 protein. However, TRIM21 can antagonize the effect of UL41 through the K48-ubiquitin-proteasome pathway, thereby decreasing the impact of UL41 on the IFN-β-JAK/STAT pathway. The figure was drawn by Figdraw (www.figdraw.com).

Together, the results of this study unravel the multifaceted mechanisms through which the PRV UL41 suppresses the antiviral responses of host cells (Figure 9). PRV UL41 inhibits the IFN-β signaling cascade by degrading the mRNA of proteins in the JAK/STAT pathway, particularly Tyk2 and STAT2. Additionally, PRV UL41 can inhibit the expression of the TRIM21 protein. However, TRIM21 can antagonize the effect of UL41 through the K48-ubiquitin-proteasome pathway, thereby decreasing the impact of UL41 on the IFN-β-JAK/STAT pathway. They offer significant leads for deciphering viral immune evasion strategies and present potential targets for developing novel antiviral strategies against PRV. Besides, the molecular mechanisms of precise interactions between PRV and host cells warrant further investigation, as they represent potential targets for novel antiviral strategies that could impact the viral life cycle and host cell defense mechanisms. In future studies, we will investigate the function of UL41 during different stages of PRV infection and its interactions with other host cellular proteins, utilizing both in vitro and in vivo models involving UL41-deficient PRV (PRV-ΔUL41). Additionally, we will evaluate the potential of UL41 as a target for antiviral intervention. In addition, our data show PRV infection upregulates IFN-α in human cells (Figure 1A), a phenomenon not directly linked to UL41 in this study. Future work should investigate whether UL41 or other PRV proteins modulate IFN-α production, for example, via TLR-dependent pathways, to understand PRV's complex regulation of the IFN response fully. These approaches aim to deepen our knowledge of UL41 and lay a scientific foundation for developing novel antiviral strategies that target UL41 in PRV, with potential applications extending to other herpesviruses more broadly.

## Data Availability

All data generated or analyzed during this study are included in this published article and its supporting files.

## Supporting Information

Supporting Information Figure S1: Expression, purification, and concentration of UL41 protein. Purified UL41 (A) and concentrated UL41 (B) were evaluated by SDS–PAGE and stained with Coomassie Brilliant Blue for visualization. Figure S2: Identification of PRV-ΔUL41 mutant viruses. (A) One-step growth curve of PRV-WT and PRV-ΔUL41. PK-15 cells were infected with 0.1 MOI of each strain. Cells and supernatants were harvested at 1, 4, 18, 36, and 48 hpi. Significance levels compared to the WT are indicated: *⁣*^*∗*^, *p*-value < 0.05; *⁣*^*∗∗*^, *p*-value < 0.01. (B) PK-15 cells were infected with PRV for 4–36 h, and the UL41 expression was determined by Western blot using anti-PRV gD antibody, anti-UL41 antibody, or anti-β-actin monoclonal antibody as the primary antibody and HRP-labeled Goat anti-mouse or rabbit IgG (H+L) antibody as the secondary antibody. (C) PK-15 cells were infected with PRV-WT and PRV-ΔUL41 (MOI = 1) for 24 h, followed by IFA. The viral gD (green) and UL41 (red) were indicated using anti-PRV gD/anti-UL41 antibodies, and the cell nucleus was stained with DAPI (blue). Scale bar, 750 μm. Mock: uninfected. Figure S3: Cytotoxicity assay of MG132. HEK293T cells were treated with different concentrations of MG132 and then assayed for survival at 12 h post-treatment. The data are presented as the means ± SD. *⁣*^*∗*^, *p*-value < 0.05; *⁣*^*∗∗*^, *p*-value < 0.01; *⁣*^*∗∗∗*^, *p*-value < 0.001. DMSO was used as the control (Con). Table S1: Primers used in this study. Table S2: Probes targeting single-stranded mRNA. Table S3: The information of UL41/VHS from herpesvirus obtained from NCBI. Table S4: Primers used to amplify the UTR of target mRNAs.
