# Supplementary material for: Pseudorabies Virus UL41 Hijacks IFN Response via JAK/STAT Pathway While Cellular TRIM21 Blocks it Through K48 Ubiquitination
Source: Transbound Emerg Dis. 2025 Nov 25;2025:3468674. doi: 10.1155/tbed/3468674 (PMC12672077; doi:10.1155/tbed/3468674)
Supplement: Supporting Information — Figure S1: Expression, purification, and concentration of UL41 protein. Purified UL41 (A) and concentrated UL41 (B) were evaluated by SDS–PAGE and stained with Coomassie Brilliant Blue for visualization. Figure S2: Identification of PRV-ΔUL41 mutant viruses. (A) One-step growth curve of PRV-WT and PRV-ΔUL41. PK-15 cells were infected with 0.1 MOI of each strain. Cells and supernatants were harvested at 1, 4, 18, 36, and 48 hpi. Significance levels compared to the WT are indicated: ⁣∗, p-value < 0.05; ⁣∗∗, p-value < 0.01. (B) PK-15 cells were infected with PRV for 4–36 h, and the UL41 expression was determined by Western blot using anti-PRV gD antibody, anti-UL41 antibody, or anti-β-actin monoclonal antibody as the primary antibody and HRP-labeled Goat anti-mouse or rabbit IgG (H+L) antibody as the secondary antibody. (C) PK-15 cells were infected with PRV-WT and PRV-ΔUL41 (MOI = 1) for 24 h, followed by IFA. The viral gD (green) and UL41 (red) were indicated using anti-PRV gD/anti-UL41 antibodies, and the cell nucleus was stained with DAPI (blue). Scale bar, 750 μm. Mock: uninfected. Figure S3: Cytotoxicity assay of MG132. HEK293T cells were treated with different concentrations of MG132 and then assayed for survival at 12 h post-treatment. The data are presented as the means ± SD. ⁣∗, p-value < 0.05; ⁣∗∗, p-value < 0.01; ⁣∗∗∗, p-value < 0.001. DMSO was used as the control (Con). Table S1: Primers used in this study. Table S2: Probes targeting single-stranded mRNA. Table S3: The information of UL41/VHS from herpesvirus obtained from NCBI. Table S4: Primers used to amplify the UTR of target mRNAs. [file 3468674.f1.pdf]

Supporting Information for

Pseudorabies Virus UL41 Hijacks IFN Response via JAK/STAT Pathway While Cellular TRIM21 Blocks It through K48 Ubiquitination

Xue Li [1, 2], Jiawei Zheng [1], Guoqing Zhang [1], Peiheng Li [1], Mengzhen Dong [1], Quan Liu [2], and Linzhu Ren [1, 3, *]
[1] College of Animal Sciences, State Key Laboratory for Diagnosis and Treatment of Severe Zoonotic Infectious Diseases, Jilin University, Changchun, China
[2] The First Hospital of Jilin University, Changchun, China.
[3] Key Lab for Zoonoses Research, Ministry of Education, Jilin University, Changchun, China
* Corresponding author: Linzhu Ren
Email: renlz@jlu.edu.cn (RL)

This file includes:

Supporting text
Fig. S1 to S3
Table S1 to S4

Supporting Information Text

Materials and methods

Plasmids construction

Recombinant plasmids were synthesized by Jinweizhi Biotechnology Co., Ltd. (Jiangsu, China). Briefly, PRV UL41 gene (GenBank: OP168821) was inserted into a pET28a, resulting in pET-28a(+)-UL41.

Plasmid transformation, expression, and purification of UL41

PRV UL41 protein was obtained as follows. Briefly, *Escherichia coli* (*E. coli*) Rosetta (DE3) competent cells (Tiangen, Beijing, China) were transformed with pET-28a(+)-UL41, and the positive bacteria were stored at −80 °C, designated as Rosetta-UL41. The Rosetta-UL41 were cultured in Luria-Bertani (LB) medium at 37 °C until reaching an optical density at 600 nm (OD_{600}) of 0.8, then treated with 1 mM isopropyl β-D-thiogalactopyranoside (IPTG, Beyotime Shanghai, China) overnight.

The IPTG-treated bacteria were centrifuged at 4 °C and 8000 rpm for 5 min and washed twice with phosphate-buffered saline (PBS, pH=8.0). The precipitate was resuspended in 10 mL of PBS (pH=8.0) containing 1 % Triton X-100 and 1 mM phenylmethylsulfonyl fluoride (PMSF, WanleiBio, Wuhan, China) and disrupted using an ultrasonic homogenizer (JY96-IIN, SCIENTZ) for 45 min on ice. The mixture was centrifuged at 12,000 rpm for 30 min at 4 °C. The supernatant was filtered through a 0.45 µm filter (Biofil, Guangzhou, China) and purified using Ni-NTA agarose (Thermo Scientific, Waltham, MA, USA). Following purification, the proteins were washed with wash buffer (5-20 mM imidazole in PBS, pH=7.4) until the absorbance at 280 nm was reduced to below 0.02, and then eluted with elution buffer (200-500 mM imidazole in PBS, pH=7.4). Then, the UL41 protein was purified using a 10 kDa ultrafiltration tube (Millipore, Billerica, MA, USA) to remove imidazole and concentrated to 1 mM, according to the manufacturer’s protocol.

The protein was analyzed using 10 % sodium dodecyl sulfate-polyacrylamide gel electrophoresis (SDS-PAGE) and stained with Coomassie Brilliant Blue for visualization.

Cytotoxicity evaluation and treatment of MG132

HEK293T cells were seeded at 5×103 cells per well (100 µL) in a 96-well plate and incubated overnight for adherence. Subsequently, cells were treated with serial dilutions of MG132 (Absin, Shanghai, China), ranging from 0.025 µM to 10 µM. Control wells were also prepared to measure background absorbance, containing all components except the cells. Following a 12 h incubation with the drug, 10 µL of CCK8 (Cell Counting Kit-8; APExBIO, Houston, TX, USA) was added to each well, and the plate was incubated for 1 h at 37°C. The absorbance of a water-soluble formazan product was then quantified using an Infinite M200 microplate spectrophotometer (Tecan) at 450 nm, which was normalized to compare with the untreated cell control. The resulting absorbance values were normalized and compared to the absorbance of the untreated cell control group to assess the cytotoxicity of MG132.

After the co-transfection of HEK293T cells with pEGFP-UL41 and pIRES-TRIM21 (porcine-derived) plasmids for 36 h, the cells were subsequently treated with MG132 at concentrations of 0.2 µM and 0.5 µM for 12 h.

Generation of the PRV UL41 Knockout strain (PRV-ΔUL41)

To generate the PRV UL41 knockout virus (PRV-ΔUL41), the sgRNA1 (5’- CACCGCACGT GAAGAACGAGACGCGGG-3’) and sgRNA2 (5’- CACCGCACTACCAGGAGACGACCGAGG-3’) were respectively inserted into the pX459V2.0-eSpCas9(1.1) vector, generating px459-KO-UL41-1 and px459-KO-UL41-2.

The Vero cells were transfected using Lipofectamine 2000 (Invitrogen, USA) with px459-KO-UL41-1/2 constructs. After 6 h, the transfected cells were infected with PRV-JL21 (PRV-WT, MOI=0.1) and selected with 2 μg/mL puromycin. After 36 h, the infected cells were harvested. Subsequently, the mutant virus (PRV-ΔUL41) was purified through two rounds of plaque purification and identified by the Western blot and Indirect immunofluorescence assay (IFA).

Indirect immunofluorescence assay (IFA)

PK-15 cells were seeded at a density of 105 per well on slides in 24-well plates. After 8 h, cells were infected with PRV-WT or PRV-ΔUL41 (MOI=1) for 24 h. Then, the cells were fixed in 80 % acetone at -80 °C overnight. After washing with PBS twice (5 min each), the cells were blocked with 3 % bovine serum albumin (BSA) for 2 h at 22-25 °C. Cells were incubated with mouse anti-PRV gD monoclonal antibody (1:100, LvduBio, China) at 22-25 °C for 2 h. After washing with PBS, the cells were incubated with anti-mouse IgG-FITC (1:500, Abbkine, Wuhan, China) in a dark box. Subsequently, in the dark box, cells were incubated with mouse anti-UL41 antibody (1:100, obtained from our lab) at 22-25 °C for 2 h, washed, and incubated with anti-mouse IgG-Cy3 (1:500, Abbkine, Wuhan, China) at 22-25 °C for 2 h. Then, the cell nucleus was stained with DAPI (2 μg/mL, Boster, Wuhan, China) for 10 min at 22-25 °C. Finally, the cells were examined using the Inverted Fluorescence microscope.

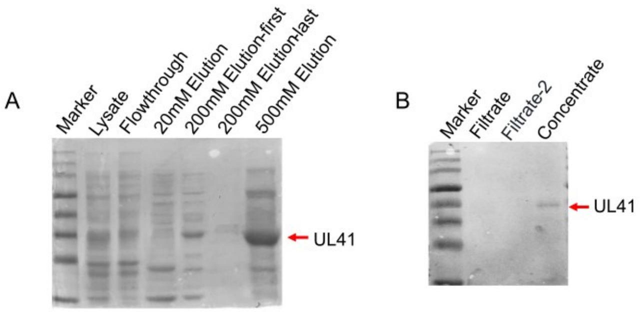

Fig. S1 Expression, purification, and concentration of UL41 protein. Purified UL41 (A) and concentrated UL41 (B) were evaluated by SDS–PAGE and stained with Coomassie Brilliant Blue for visualization.

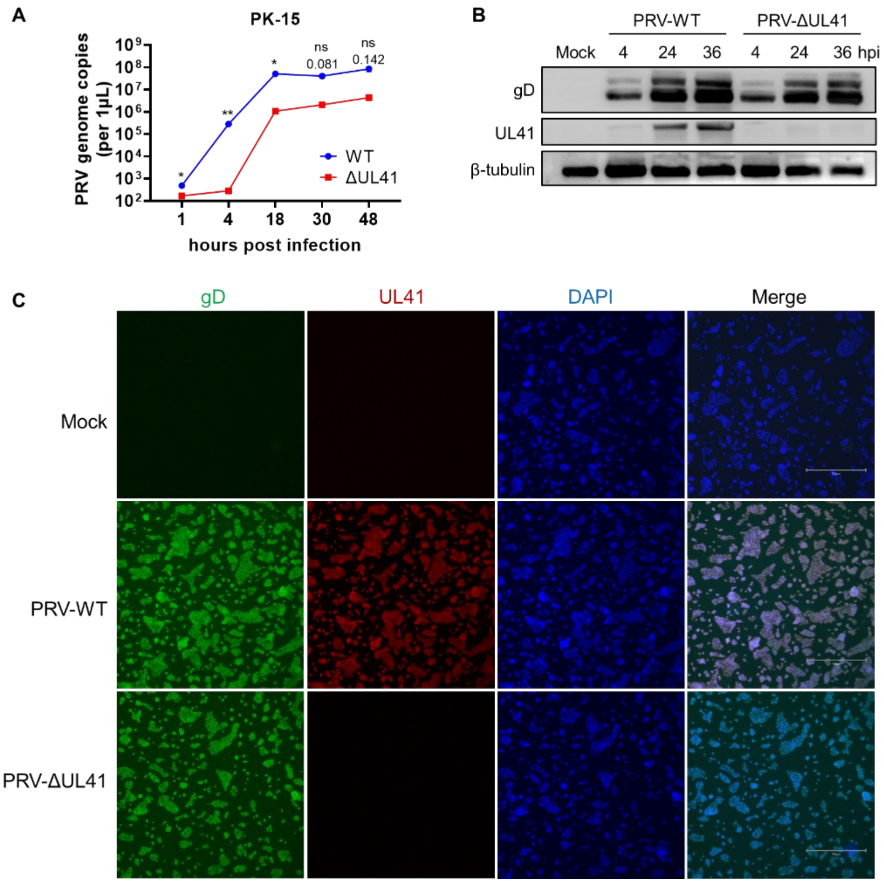

Fig. S2 Identification of PRV-ΔUL41 mutant viruses. (A) One-step growth curve of PRV-WT and PRV-ΔUL41. PK-15 cells were infected with 0.1 MOI of each strain. Cells and supernatants were harvested at 1, 4, 18, 36, and 48 hpi. Significance levels compared to the WT are indicated: *, p-value < 0.05; **, p-value < 0.01. (B) PK-15 cells were infected with PRV for 4-36 h, and the UL41 expression was determined by Western blot using anti-PRV gD antibody, anti-UL41 antibody, or anti-β-actin monoclonal antibody as the primary antibody and HRP-labeled Goat anti-mouse or rabbit IgG (H+L) antibody as the secondary antibody. (C) PK-15 cells were infected

with PRV-WT and PRV-ΔUL41 (MOI=1) for 24 h, followed by IFA. The viral gD (green) and UL41 (red) were indicated using anti-PRV gD/anti-UL41 antibodies, and the cell nucleus was stained with DAPI (blue). Scale bar, 750 μm. Mock: uninfected.

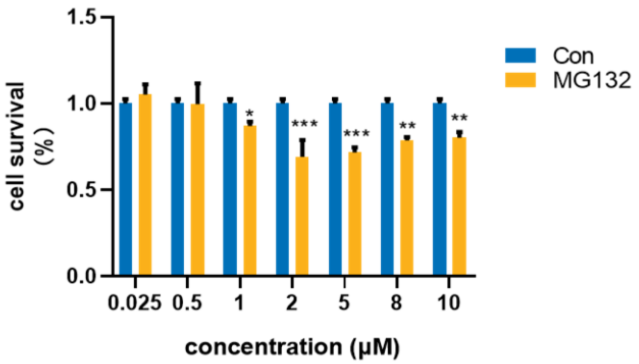

Fig. S3 Cytotoxicity assay of MG132. HEK293T cells were treated with different concentrations of MG132 and then assayed for survival at 12 h post-treatment. The data are presented as the means ± SD. *, p-value < 0.05; **, p-value < 0.01; ***, p-value < 0.001. DMSO was used as the control (Con).

Table S1. Primers used in this study

Primers	Sequence (5'-3')	GenBank No.	Reference
PRV-F	GGTTCAACGAGGGCCAGTACCG	OR195723	(1)
PRV-R	GCGTCAGGAATCGCATCACGT		
PRV qUL41-F	AACAAGGTCTTTGGCTCGCT	OP168821	This study
PRV qUL41-R	TATGGGCGTTACAGTCGTCC		
human GAPDH-F	TGGAAATCCCATCACCATCT	NM_002046	(2)
human GAPDH-R	GTCTTCTGGGTGGCAGTGAT		
human IFNA-F	GCTTGGGATGAGACCCTCCTA	NM_024013	Primer bank
human IFNA-R	CCCACCCCCTGTATCACAC		
human IFNB1-F	ATGACCAACAAGTGTCTCCTCC	NM_002176	Primer bank
human IFNB1-R	GGAATCCAAGCAAGTTGTAGCTC		
human cGAS-F	ACGTGCTGTGAAAACAAAGAAG	NM_138441	(2)
human cGAS-R	GTCCCACTGACTGTCTTGAGG		
human sting-F	AGCATTACAACAACCTGCTACG	NM_198282	Primer bank
human sting-R	GTTGGGGTCAGCCATACTCAG		
human TBK1-F	GCTAGCATGGCTAAGGCAAT	NM_013254	This study
human TBK1-R	TTCAATCAGCCATCGTATCCC		
human IRF3-F	AGAGGCTCGTGATGGTCAAG	NM_00119712 8	Primer bank
human IRF3-R	AGGTCCACAGTATTCTCCAGG		
human JAK1-F	CTTTGCCCTGTATGACGAGAAC	NM_002227	Primer bank
human JAK1-R	ACCTCATCCGGTAGTGGAGC		
human TYK2-F	GGAGGAGGGTTCTAGTGGCA	NM_003331	Primer bank
human TYK2-R	ATGTCCCGGAAGTCACAGAAG		
human STAT1-F	ATCAGGCTCAGTCGGGGAATA	NM_007315	Primer bank
human STAT1-R	TGGTCTCGTGTTCTCTGTTCT		
human STAT2-F	GAGCCAGCAACATGAGATTGA	NM_198332	Primer bank
human STAT2-R	GCCTGGATCTTATATCGGAAGCA		
human IRF9-F	GCCCTACAAGGTGTATCAGTTG	NM_006084	Primer bank
human IRF9-R	TGCTGTCGCTTTGATGGTACT		
human ISG15-F	TTCGTCGCATTTGTCCACCA	NM_005101	Primer bank
human ISG15-R	CGCAGATCACCCAGAAGATCG		
hISG56-F	AGAAGCAGGCAATCACAGAAAA	NM_001548	Primer bank
hISG56-R	CTGAAACCGACCATAGTGGAAAT		
hIFITM1-F	CCAAGGTCCACCGTGATTAAC	NM_003641	Primer bank
hIFITM1-R	ACCAGTTCAAGAAGAGGGTGTT		
human IFITM3-F	CATGTCGTCTGGTCCCTGTTCAAC	NM_021034	This study
human IFITM3-R	CTTCACGGAGTAGGCGAATGCTATG		
human SOCS1-F	TTTTCGCCCTTAGCGTGAAGA	NM_003745	Primer bank
human SOCS1-R	GAGGCAGTCGAAGCTCTCG		
human TRIM21-F	GTCCTGGAAAGGAGTGAGTCC	NM_003141	Primer bank
human TRIM21-R	CTGAAAGTATCAGCCACGGATT		
pig GAPDH-F	gccatcaccatcttccagg	NM_002046	(1)

pig GAPDH-R	tcacgcccatcacaaacat		
pig JAK1-F	CCAGGCAAGAGTGCATAGAA	NM_214114	(1)
pig JAK1-R	GTGGTTCCAAAGCTCCATTTG		
pig Tyk2-F	TGCTATGACCCGACCAACGA	NM_001114670	this study
pig Tyk2-R	TCGCAGCAGCCCTTGTACTT		
pig STAT1-F	TTTGGGCACTCACACGAAGG	NM_213769	this study
pig STAT1-R	GGCTGGCACAATTGGGTTTC		
pig STAT2-F	ATCGGACCAGACAGGAGCAA	NM_213889	this study
pig STAT2-R	GGCTCCAATGCAGGCTTTCT		
pig IRF9-F	GGAACTGGGTGGTGGAGCAA	NM_001078670	this study
pig IRF9-R	CTCTTGTTGAGGGCACAGCG		
pig TRIM21-F	CCGTCTCCTTCTACAACATCAG	NM_001163649	(3)
pig TRIM21-R	GCATTTCTTCCACCGTCATTG		
UL41 C132A-F	GCACAGACTGGCTGTGAACCTGATCAGACACATG	OP168821	this study
UL41 C132A-R	ACGATTCTAGGGGCGTGC		
UL41 F78A-F	CTTCCCCATCgctGTGAGCGACAG	OP168821	this study
UL41 F78A-R	TAGGTTCTCTTGTGCAGC		
TRIM21-ΔSPRY/PRY-F	CACCACCACTAGGAATTCTGCAGATATCCAGCAC	NM_001163649	this study
TRIM21-ΔSPRY/PRY-R	ATGATGATGCCAGCCCGTCCTCAGTGG		
TRIM21-ΔRING-F	CGGCAGCACTTCCTGCTT	NM_001163649	this study
TRIM21-ΔRING-R	CGTGACCTCATCCCACATC		

Table S2. Probes targeting single-stranded mRNA.

RNA Probes	Sequence（5'-3'）
IRF9-1	GCGACAGCCUGGACAGCAAC
IRF9-2	GGGCAGUUUCCCGGAGUGUG
IRF9-3	GGAUUCCCUGGAAACAUGCA
IRF9-4	UUUAUAUUUUCCUCUUAGAU
Tyk2-1	CAGGAAGAAGCCGCGGGGAC
Tyk2-2	CGAUGGGGAAACUGAGGCCC
Tyk2-3	CACCUGCCGGGAGCAUGCCU
Tyk2-4	CCUGCUCCAGGAUGAAACCA
Tyk2-5	CUUAUUUUUCUUUCUUGGCC
Tyk2-6	GGUAAUAAACUCAUGUUUUCUCU
STAT2-1	UCAGCCCUUUUCCCAGGAU
STAT2-2	CUUGCUUUCUUCUUUCCUUUACU
STAT2-3	GGUGGCUGUUUCUUGUCUGUUA

STAT2-4	GGGCGCGGGGACUGCAACCC
STAT2-5	GGUUGUGGACCUGGACAAAG

Note: the conserved sequences corresponding to KTTTTCY and CSDGGA were labeled in red.

Table S3. The information of UL41/VHS from herpesvirus obtained from NCBI.

Accession	Length (aa)	organism name	common name	species	Host
WEU66586	365	Suid alphaherpesvirus 1	PRV	Varicellovirus suidalpha1	Sus scrofa
YP_009137116.	489	Human alphaherpesvirus 1	HSV1	Simplexvirus humanalpha1	Homo sapiens
YP_009137193	492	Human alphaherpesvirus 2	HSV2	Simplexvirus humanalpha2	Homo sapiens
NP_040140	455	Human alphaherpesvirus 3	HSV3	Varicellovirus humanalpha3	Homo sapiens
YP_010374206	459	Bovine herpesvirus type 1	BHV1	Varicellovirus bovinealpha1	Bos taurus
YP_053064	497	Equid alphaherpesvirus 1	EHV1	Varicellovirus equidalpha1	Equus caballus
YP_010795059	496	Equid alphaherpesvirus 8	AHV3	Varicellovirus equidalpha8	Equus caballus
YP_182370	398	Gallid alphaherpesvirus 1	GaHV1	Iltovirus gallidalpha1	Gallus gallus
YP_001033970	441	Gallid alphaherpesvirus 2	GaHV2	Mardivirus gallidalpha2	Gallus gallus
YP_001129390	486	Human gammaherpesvirus 8	HSV8	Rhadinovirus humangamma8	Homo sapiens

Table S4. Primers used to amplify the UTR of target mRNAs.

Primers	Sequence (5’-3’)	Reference
Tyk2-5’F	TCCGGGTTCGAGCTTGTGTT	this study
Tyk2-5‘R	TCCAAGTGCAGCCTGTCAAG	
Tyk2-3‘-1F	TTGGCCGTGAGCCTAACCAT	this study
Tyk2-3‘-1R	TGGTGGGCCTCAAGTTTGGA	
Tyk2-3‘-2F	CCTGCTCCAGGATGAAACCA	this study
Tyk2-3‘-2R	TAGGCTCACGGCCAAGAAAG	
STAT2-5'-F	TGCAACCCTAATCAGAGCCCA	this study
STAT2-5'-R	GCTGTGCGAGTAAAGCTGGT	
STAT2-3'-1F	TCAGGCATGTGTCCCTTCCA	this study
STAT2-3'-1R	ACCTATGGCTCAGCATCTGTTCT	
STAT2-3'-2F	TCGTTGGTTGTGGACCTGGA	this study
STAT2-3'-2R	CCCTGGGAATAGCTAAGGTGTGA	
IRF9-5'-F	CAGGAGTTAAGCTGAGGTCG	this study
IRF9-5'-R	CGGAACATGGTCTTAGCTGT	
IRF9-3'-F	TGGTGGAGAACTCAAGGCTAA	this study
IRF9-3'-R	TTAGAGTTGGGAGGTCAGGGA	

SI References

1. X. Li *et al.*, Coinfection of Porcine Circovirus 2 and Pseudorabies Virus Enhances Immunosuppression and Inflammation through NF-κB, JAK/STAT, MAPK, and NLRP3 Pathways. *Int J Mol Sci* **23** (2022).
2. N. Kerur *et al.*, cGAS drives noncanonical-inflammasome activation in age-related macular degeneration. *Nature medicine* **24**, 50-61 (2018).
3. L. Yang *et al.*, Porcine TRIM21 Enhances Porcine Circovirus 2 Infection and Host Immune Responses, But Inhibits Apoptosis of PCV2-Infected Cells. *Viruses* **14** (2022).
